# Nonlinear association between TyG-related Indices and motoric cognitive risk syndrome in depressive individuals: evidence from the CHARLS study

**DOI:** 10.3389/fpsyt.2025.1622973

**Published:** 2025-08-28

**Authors:** Ze-kun Wei, Cun-yang Li, Zhi-yun Liu, Bo-lin Wang, Can Wang, Yang Liu, Te-jin Ba, Li Kong, Fei-hu Zhang

**Affiliations:** ^1^ The First Clinical Medical College, Shandong University of Traditional Chinese Medicine, Jinan, China; ^2^ Department of Emergency Center, Shandong University of Traditional Chinese Medicine Affiliated Hospital, Jinan, China; ^3^ Department of Emergency and Critical Care Medicine, International Mongolian Medical Hospital of Inner Mongolia Autonomous Region, Hohhot, China

**Keywords:** motoric cognitive risk syndrome, TyG-BMI, insulin resistance, nonlinear association, depression, CHARLS

## Abstract

**Background:**

Motoric Cognitive Risk Syndrome (MCR), defined by cognitive complaints and slow gait, is a pre-dementia condition linked to metabolic dysfunction. The triglyceride-glucose (TyG) index and its composite derivatives are surrogate markers of insulin resistance and may contribute to cognitive decline. This study investigated the associations between TyG-related indices and MCR risk in middle-aged and older adults with depressive symptoms, focusing on nonlinear relationships and subgroup modifications.

**Methods:**

This study included 5,657 participants aged ≥45 years from the China Health and Retirement Longitudinal Study (CHARLS). Associations between four TyG-related indices (TyG, TyG-BMI, TyG-WC, TyG-WHtR) and MCR were assessed using logistic regression. Nonlinear associations were examined via generalized additive models and two-piecewise linear regression. Stratified analyses explored effect modifications by age, sex, education, and depressive status.

**Results:**

All TyG-related indices were positively associated with MCR. TyG-BMI demonstrated the strongest and most consistent association, with a significant threshold at 276.05. Below this point, MCR risk increased with TyG-BMI (OR = 1.01; 95% CI: 1.004-1.017; P = 0.003), while no association was found above it (P = 0.416). Similar nonlinear trends were observed in depressive individuals. Subgroup analyses indicated stronger associations in older adults (≥60 years).

**Conclusion:**

TyG-BMI demonstrates a nonlinear association with MCR risk and may serve as an accessible biomarker for early cognitive risk detection in depressive individuals.

## 1 Introduction

Motoric Cognitive Risk Syndrome (MCR), defined by the co-occurrence of slow gait and cognitive complaints, was first conceptualized by Verghese et al. as a clinical phenotype to identify individuals at elevated risk for dementia (1). As an extension of the mild cognitive impairment (MCI) construct, MCR expands early diagnostic frameworks by integrating both cognitive and motor domains (2). An increasing body of evidence has linked MCR with various comorbidities including diabetes, cerebrovascular disease, and depression (3, 4). Aliberti et al. observed that MCR was particularly associated with cognitive deficits among middle-aged individuals exhibiting depressive symptoms (5). Furthermore, systematic reviews by Kim and Fanelli emphasized the central role of insulin resistance (IR) in neurocognitive dysfunction, highlighting the relevance of metabolic disturbances in cognitive aging (6, 7).

The TyG index is a validated surrogate measure for insulin resistance, first developed by Simental-Mendía and Guerrero-Romero (8, 9). It has shown predictive value for a broad range of outcomes, including cognitive impairment (10), cardiovascular events (11), and hypertension (12). Notably, recent studies suggest that TyG outperforms traditional indices such as HOMA-IR in detecting IR, underscoring its clinical utility (13). Meta-analyses have confirmed the association of TyG with progression of multiple metabolic diseases, including nonalcoholic fatty liver disease and chronic kidney disease (14–16).

In parallel, obesity has emerged as a critical contributor to both metabolic dysregulation and neuropsychiatric conditions. Its link with depression is particularly well-established, mediated through pathways involving hypothalamic-pituitary-adrenal (HPA) axis dysfunction, systemic inflammation, and altered cortisol secretion (17–21). The 2024 UK Millennium Cohort Study revealed that elevated BMI was associated with increased body dissatisfaction and depressive symptoms, particularly in adolescent females (22–24). Furthermore, longitudinal data have demonstrated a U-shaped association between BMI and depression risk, suggesting that both under- and overweight states can adversely affect mental health (25).

Given this background, TyG-BMI—a composite indicator integrating both insulin resistance and obesity—may represent a more comprehensive marker of metabolic risk relevant to cognitive decline. This study therefore investigates the associations between TyG-related indices and MCR in a nationally representative sample of Chinese middle-aged and older adults with depressive symptoms. By employing nonlinear regression models and stratified analyses, this work seeks to elucidate not only whether such associations exist, but also how they vary across population subgroups. The findings have the potential to inform the development of targeted screening strategies and early interventions for cognitive deterioration.

Against the backdrop of an accelerating global aging population, depressed elderly people have become a key group in the primary prevention of dementia. Showed that insulin resistance (IR) in the literature as the key metabolic abnormalities, can through the metabolism - blood vessels - multiple cascade inflammation (26), It promotes the synergistic deterioration of Aβ/tau pathology and neural network dysfunction (27), resulting in dual damage to the cognitive-motor network and ultimately developing into MCR. Community research shows that MCR accounts for 10% of the elderly, and the risk of dementia increases by 3 to 5 times within 3 to 5 years, becoming the most easily detectable precursor signal. The TyG index, an accessible IR indicator, has been associated with cognitive decline and AD pathology in the non-depressed population, but its longitudinal association with MCR has not yet been verified in the depressed and high-IR population.

Triglyceride-glucose-related indicators, as inexpensive alternative indicators of insulin resistance, provide practical screening tools for areas with limited resources. However, it remains unclear whether TyG-BMI has a threshold effect on the risk of MCR in elderly people with depression. Clarifying this relationship will provide relevant references for this high-risk yet underserved group.

## 2 Methods

### 2.1 Data source

Data were obtained from the China Health and Retirement Longitudinal Study (CHARLS), a nationally representative longitudinal survey that collects a wide range of demographic, socioeconomic, and health-related data on individuals aged ≥45 years across China. CHARLS is a publicly available dataset and approved for secondary analysis. The current analysis was based on the 2015 wave of CHARLS. The study was approved by the Institutional Review Board of Peking University (IRB00001052-11015), and written informed consent was obtained from all participants. All data were collected using face-to-face, computer-assisted individual interviews (CAPI). The CHARLS project team recruited undergraduate and graduate students as field interviewers (28–30).All interviewers received systematic interview skills training and passed the assessment, and then qualified in the simulation drill before they could work. The CAPI program can prompt logic errors in the field and allow correction on the spot to ensure data quality. At the same time, the project team assigned an experienced supervisor to supervise the whole site work and solve any investigation and implementation problems in a timely manner. Further details of the sampling design, questionnaire content, follow-up procedures, and quality control have been published elsewhere (28).

### 2.2 Study population

A cross-sectional analysis was conducted using data from 21,095 participants in the 2015 CHARLS database. A total of 5,657 participants were ultimately included after excluding 14,052 individuals due to missing cognitive questionnaire or other essential information, 244 without demographic or with extreme values (e.g., BMI/waist circumference), 8 with incomplete depression scale data, and 1,134 lacking TyG and related biomarker information.

**Figure 1:**
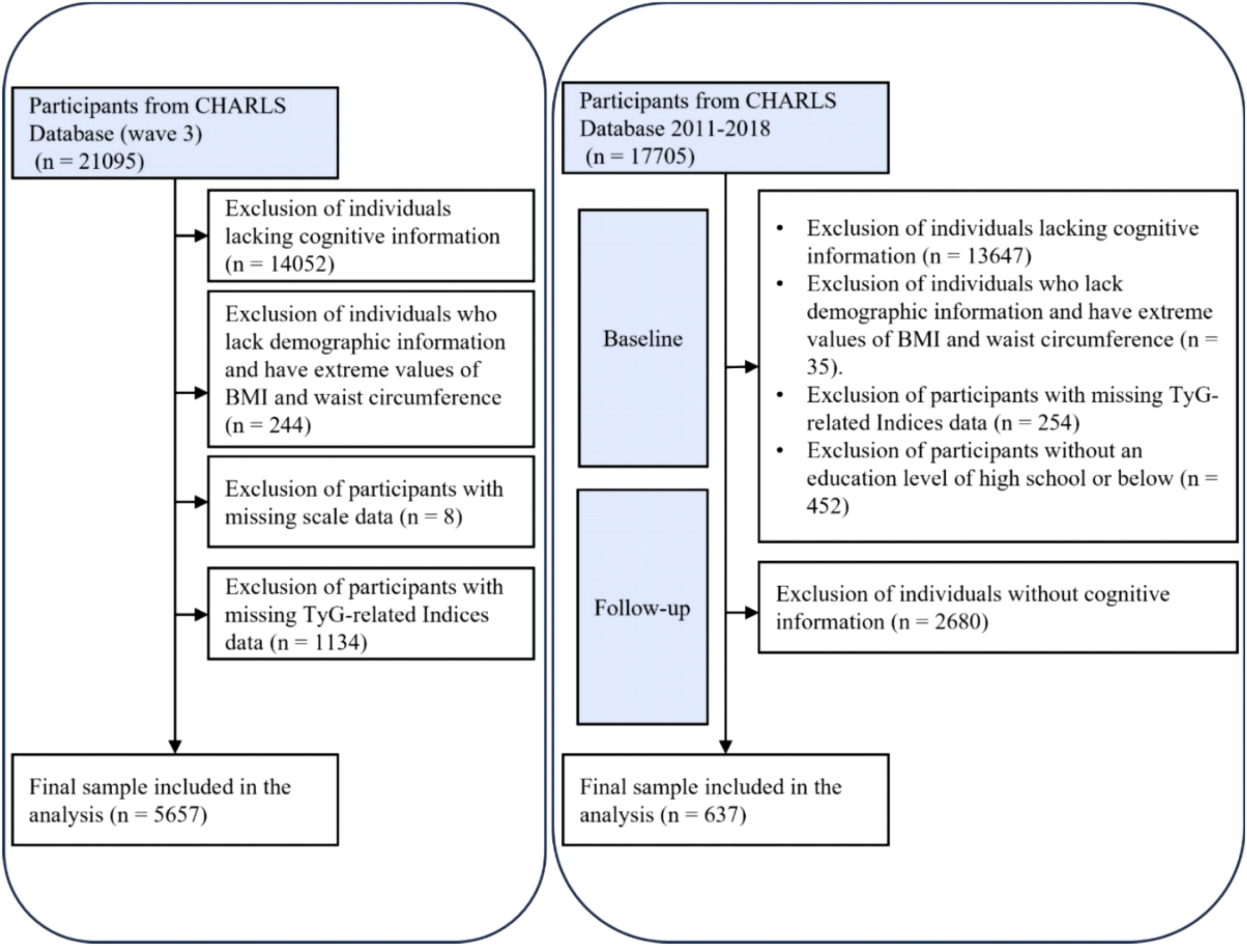
Flowchart.

For sensitivity analysis, a cohort study was performed based on the initial wave of CHARLS interviews, which included 17,705 participants at baseline. Following the exclusion of 13,647 individuals without cognitive data, 35 without demographic or with extreme values (e.g., BMI/waist circumference), 254 missing TyG data, 452 with education levels exceeding high school, and 2,680 with missing follow-up cognitive assessments, a final sample of 637 participants was retained (**Figure 1**).

### 2.3 Selection of variables

#### 2.3.1 Definition of TyG-related Indices

1. TyG Index: ln((fasting triglycerides (mg/dL) × fasting glucose (mg/dL))/2) (31).
2. BMI: Body mass index, calculated as weight (kg) divided by height squared (m²) (32).
3. TyG-WC: TyG index × waist circumference (cm) (33).
4. TyG-WHtR: TyG index × waist-to-height ratio (waist circumference (cm)/height (cm)) (34).
5. TyG-BMI: TyG index × BMI (35).

#### 2.3.2 Motoric Cognitive Risk Syndrome

According to previous studies, MCR is defined as the presence of cognitive complaint and slow gait but without dementia. To assess subjective cognitive complaints, self-reported questions about memory loss and cognitive measurement scales were adopted in the study. The specific question is: “How do you evaluate your memory now?” The answer options include: excellent, very good, good, average or poor. Participants who answered “average” or “poor” were recorded as having cognitive problems (36). In the CHARLS study, self-reported memory states have been proven reliable.

The CHARLS research also developed a questionnaire to assess executive function and episodic memory, two cognitive abilities. Words’ immediate and delayed recall serve as measures of episodic memory. The researchers read ten separate Chinese words to each participant. Two measures of memory performance were administered: the number of words correctly remembered right away (immediate word recall score) and the number of words correctly remembered four minutes later (delayed word recall score). The sum of the scores for both immediate and delayed word recall is used to determine the episodic memory score, which can be anything from zero to ten. Scores ranged from 0 to 11, and they were derived from two sources: cognitive state telephone interviews (TICS) and the ability to graphically depict mental state problems. When combined, the scores for executive function and episodic memory make up the cognitive function score. The cognitive function is evaluated from 0 to 21, with higher scores indicating better performance. Cognitive impairment is defined as a total score below 6 (37). Therefore, the inclusion criteria for cognitive impairment in this study included: participants who answered “How do you currently evaluate your memory” as “average” or “poor”, as well as those with a total cognitive function score lower than 6.

Slow gait is defined as: less than 0.44 m/s for men under 75 years old, less than 0.35 m/s for men 75 years and above, less than 0.41 m/s for women under 75 years old, and less than 0.33 m/s for women 75 years and above (36).

Previous literature indicates that many traditional cognitive tests (such as those requiring tasks like reading, writing, and calculation) have limited applicability to people with low educational levels (such as those without formal education) (38). However, there may be a “ceiling effect” (high education masking cognitive impairment) in the high school and above group (39), and the high school and below group is more common in the distribution of education, especially with a higher proportion among the middle-aged and elderly population (40). Therefore, in the sensitivity analysis of cohort studies, people with a high school education level or below were selected as the subjects, taking into account both measurement validity and representativeness.

#### 2.3.3 Depressive symptoms

Depressive symptoms are assessed through the 10 questions of the Center for Epidemiological Studies Depression Scale (CES-D10). Subjects are required to answer questions about their emotions and behaviors based on the situation in the past week. The total score ranges from 0 to 30, with higher scores indicating more severe depressive symptoms (41).

#### 2.3.4 Covariates

Demographic and behavioral covariates included age, sex, place of residence (urban/rural), smoking status (yes/no), and alcohol consumption (yes/no) (28, 29, 42).

### 2.4 Statistical analysis

All analyses were conducted using R version 4.2.0 and Empower (R) (www.empowerstats.com, X&Y Solutions, Inc. Boston, MA, USA). Continuous variables were summarized as mean ± standard deviation (SD) and compared using t-tests. Categorical variables were presented as percentages, with group comparisons performed using chi-square tests.

Logistic regression models were used to assess associations between TyG-related indices and MCR:

1. Model I: unadjusted.
2. Model II: adjusted for age, sex, and residence.
3. Model III: further adjusted for education, smoking, and drinking status.

To explore the potential nonlinear associations between the TYG index and MCR in the population with depressive symptoms, generalized additive models (GAMs) and piecewise linear regression analyses were applied. Threshold effects were evaluated using a two-piecewise regression model, with inflection points determined via log-likelihood ratio tests. Subgroup analyses were performed among the depressed population to examine effect modification by demographic and behavioral variables including age group, education level, alcohol use, smoking, sex, depression. Additionally, a sensitivity analysis was conducted using the E-value method to assess the impact of unmeasured confounding factors in the study.

## 3 Results

### 3.1 Baseline characteristics

Cohort participants (n=637) were younger (65.1 ± 4.91 vs 67.21 ± 6.41 years) and more often male (68.76% vs 49.76%). TyG (8.69 ± 0.64 vs 8.70 ± 0.63), TyG-BMI (203.3 ± 39.3 vs 205.4 ± 42.4), TyG-WC (745.4 ± 117.2 vs 752.2 ± 122.5) and TyG-WHtR (4.68 ± 0.76 vs 4.80 ± 0.81) were similar. Rural residence was reported by 61.38% and 63.50%; current smoking by 51.65% and 47.59%; current drinking by 49.92% and 46.56%. Depression was present in 29.83% vs 33.94%, and motoric cognitive risk syndrome in 4.87% vs 2.69% (**Table 1**).

**Table 1:** Baseline characteristics of participants by MCR status.

Variable	Cohort study	Cross-sectional study
Mean+SD		
Age	65.09 ± 4.91	67.21 ± 6.41
TyG-WHtR	4.68 ± 0.76	4.80 ± 0.81
TyG-WC	745.35 ± 117.23	752.16 ± 122.54
TyG	8.69 ± 0.64	8.70 ± 0.63
TyG-BMI	203.29 ± 39.26	205.44 ± 42.41
N (%)		
Gender		
woman	199 (31.24%)	2842 (50.24%)
man	438 (68.76%)	2815 (49.76%)
Living status		
Urban Community	246 (38.62%)	2065 (36.50%)
Rural Village	391 (61.38%)	3592 (63.50%)
Smoke status		
No	308 (48.35%)	2965 (52.41%)
Yes	329 (51.65%)	2692 (47.59%)
Drink status		
No	319 (50.08%)	3023 (53.44%)
Yes	318 (49.92%)	2634 (46.56%)
Depression		
No	447 (70.17%)	3737 (66.06%)
Yes	190 (29.83%)	1920 (33.94%)
Motoric cognitive risk syndrome		
No	606 (95.13%)	5505 (97.31%)
Yes	31 (4.87%)	152 (2.69%)

### 3.2 Associations between TyG-related indices and MCR

In the cross-sectional study, all four TyG-related measures were positively associated with CI in the fully adjusted covariate model. The results of **Table 2** showed that the OR of TyG-BMI was 1.005 (95%CI 1.001-1.008, *P* = 0.004); The OR of TyG-WHtR was 1.535 (1.256-1.875, *P* < 0.001). The OR of TyG-WC was 1.002 (1.001-1.004, *P* < 0.001). The OR of TyG was 1.313 (1.017-1.694, *P* = 0.036). Trends were further confirmed by quartile comparisons. These findings suggest that higher levels of these indicators are associated with greater risk of MCR, and this association remains robust after adjustment covariate.

**Table 2:** Logistic regression analysis of the relationship between TyG-related index and MCR(cross-sectional study).

Exposure	Non-adjusted	Adjust I	Adjust II
TyG-BMI	1.004 (1.001, 1.007) 0.025	1.005 (1.001, 1.008) 0.005	1.005 (1.001, 1.008) 0.004
TyG-BMI quartile			
Q1	1	1	1
Q2	0.969 (0.592, 1.585) 0.900	1.082 (0.658, 1.781) 0.755	1.094 (0.665, 1.801) 0.723
Q3	1.031 (0.635, 1.674) 0.902	1.212 (0.735, 1.997) 0.451	1.235 (0.749, 2.036) 0.409
Q4	1.628 (1.048, 2.531) 0.030	1.989 (1.247, 3.173) 0.004	2.015 (1.263, 3.217) 0.003
*p* for trend	0.02333	0.00303	0.00250
TyG-WHtR	1.605 (1.332, 1.935) <0.001	1.533 (1.254, 1.875) <0.001	1.535 (1.256, 1.875) <0.001
TyG-WHtR quartile			
Q1	1	1	1
Q2	1.000 (0.578, 1.731) 1.000	0.979 (0.562, 1.703) 0.939	0.997 (0.572, 1.735) 0.991
Q3	1.674 (1.023, 2.740) 0.040	1.633 (0.979, 2.722) 0.060	1.663 (0.997, 2.774) 0.051
Q4	2.241 (1.401, 3.584) <0.001	2.045 (1.232, 3.394) 0.006	2.071 (1.248, 3.437) 0.005
*p* for trend	0.00007	0.00094	0.00082
TyG-WC	1.002 (1.001, 1.003) <0.001	1.002 (1.001, 1.004) <0.001	1.002 (1.001, 1.004) <0.001
TyG-WC quartile			
Q1	1	1	1
Q2	0.934 (0.560, 1.558) 0.794	0.999 (0.596, 1.673) 0.996	1.013 (0.604, 1.699) 0.961
Q3	1.099 (0.672, 1.798) 0.707	1.154 (0.699, 1.905) 0.575	1.172 (0.710, 1.937) 0.535
Q4	1.907 (1.225, 2.968) 0.004	2.095 (1.323, 3.317) 0.002	2.115 (1.336, 3.351) 0.001
*p* for trend	0.00171	0.00080	0.00070
TyG	1.307 (1.026, 1.663) 0.030	1.314 (1.018, 1.696) 0.036	1.313 (1.017, 1.694) 0.036
TYG quartile			
Q1	1	1	1
Q2	0.911 (0.557, 1.491) 0.711	0.870 (0.529, 1.431) 0.584	0.869 (0.529, 1.430) 0.582
Q3	1.029 (0.638, 1.659) 0.908	0.959 (0.590, 1.560) 0.867	0.961 (0.591, 1.564) 0.873
Q4	1.548 (0.999, 2.401) 0.051	1.519 (0.963, 2.395) 0.072	1.517 (0.962, 2.392) 0.073
*p* for trend	0.03413	0.04997	0.05004

The longitudinal cohort included 637 participants without MCR at baseline who had at least three follow-up complete MCR and the mental state question component from the telephone cognitive status interview (TICS) as well as complete data for graphical mapping. Adjustment for the same covariates as in the cross-sectional analysis showed similar trends for TyG-BMI, with an odds ratio of 1.019 (95% CI: 1.004-1.034, *P* = 0.013) in a model with full covariate adjustment; the results are shown in **Supplementary Table 1**. the same analysis was conducted in participants without depressive symptoms within the same cohort, and the association between TyG-BMI and MCR was no longer significant, which underscores the specificity of our findings to the depressed subgroup (**Supplementary Table 2**).

The E-value was calculated for the primary association (OR=1.005) between TyG-BMI and MCR as a sensitivity analysis. The results showed that an unmeasured confounding factor with a relative risk of ≥1.08 for both exposure and outcome was required to fully explain the currently observed association. This threshold is much higher than the effect size of common lifestyle variables (physical activity, dietary habits) (43, 44), suggesting that unmeasured confounding has a limited impact on the core conclusion.

### 3.3 Nonlinear relationship and threshold effect

**Figure 2:**
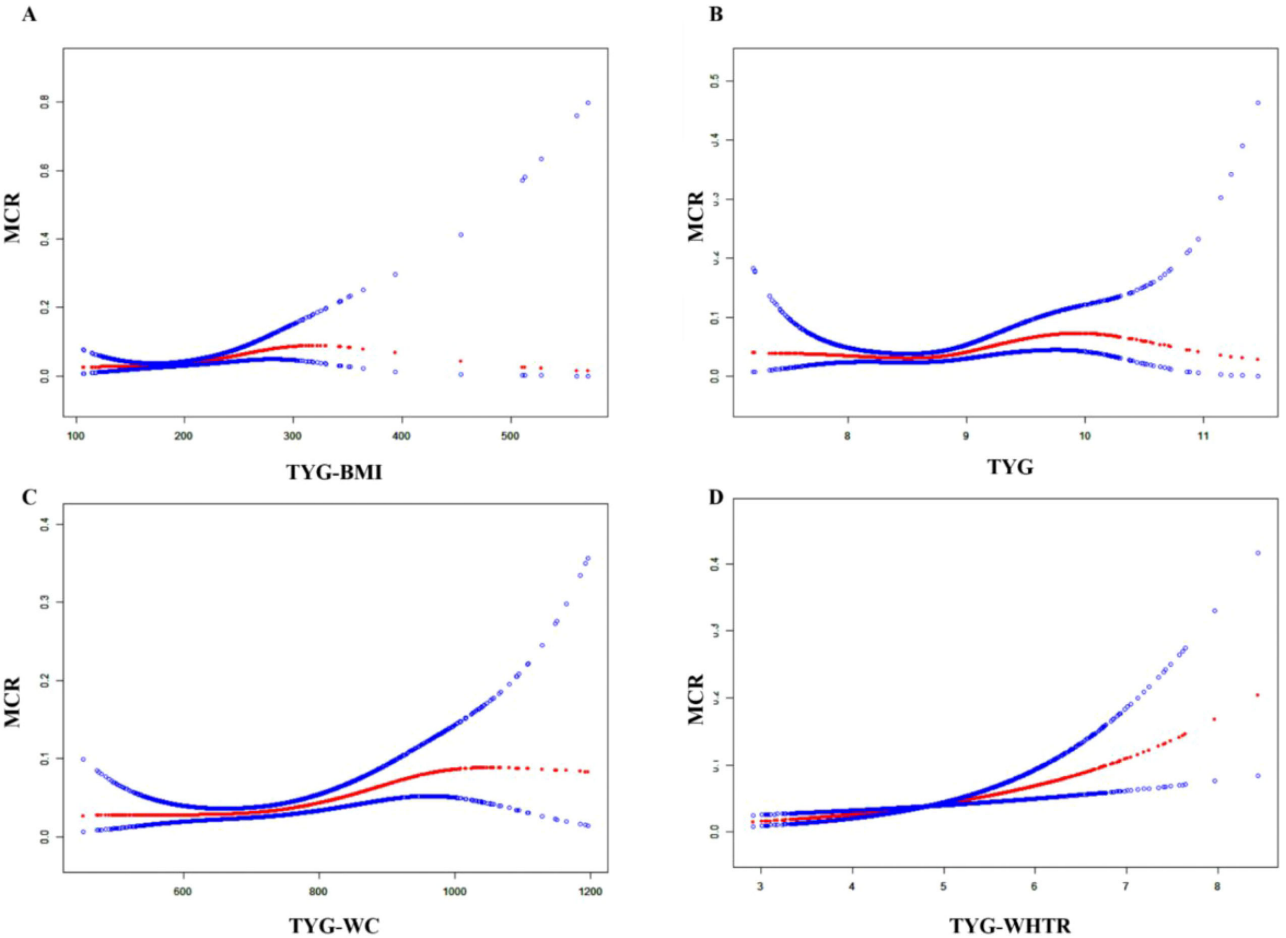
The nonlinear associations between TyG-BMI **(A)**, TyG **(B)**, TyG-WC **(C)**, and TyG-WHtR **(D)** and MCR in the population with depression.

In the middle-aged and elderly population with depression, TyG-BMI, TyG-WHtR, TyG-WC and TyG all show nonlinear threshold associations. TyG-BMI<276.05 is a risk factor for MCR. TyG-WHtR, TyG, and TyG-WC show non-linear relationships. (**Table 3** and **Figure 2**). The results of the cohort study also showed a nonlinear threshold association of TyG-BMI. When TyG-BMI > 190.155, the risk of developing MCR in the depressed population significantly increased (OR=1.033, 95CI:1.013-1.054, *P*= 0.001) (**Supplementary Table 3**).

**Table 3:** Threshold effect analysis of TyG-BMI and TyG on MCR risk in depressed population.

Outcome	TyG-BMI	TyG-WHtR	TyG-WC	TyG
Model I	1.005 (1.001, 1.010) 0.0179	1.668 (1.256, 2.214) 0.0004	1.003 (1.001, 1.005) 0.0019	1.456 (1.022, 2.075) 0.0374
Model II				
Inflection point	276.05	4.276	959.895	8.614
< Inflection point	1.010 (1.004, 1.017) 0.0028	0.722 (0.217, 2.399) 0.5950	1.004 (1.001, 1.006) 0.0012	0.732 (0.279, 1.921) 0.5262
> Inflection point	0.992 (0.971, 1.014) 0.4776	1.889 (1.356, 2.631) 0.0002	0.996 (0.983, 1.009) 0.5354	1.893 (1.166, 3.074) 0.0098
Log-likelihood Tatio test	0.039	0.184	0.207	0.150

In this subgroup analysis, the association between TYG-related indicators and outcome risk was consistent across different populations. Regarding gender, the associations of TyG-WHtR (OR=1.523) and TyG-WC (OR=1.002) with MCR were significant in women. For individuals aged ≥60 years, the associations of all indicators were stronger than in those aged <60 years, especially for TyG-WHtR (OR=1.546) and TyG (OR=1.285), which reached statistical significance. There were minimal differences based on place of residence. Among smokers, the effect of TyG-BMI (OR=1.006) was significant, while in non-smokers, the association of TyG-WHtR (OR=1.553) was stronger. In drinkers, the associations of TyG-BMI (OR=1.008) and TyG-WHtR (OR=1.692) were significant. Overall, the associations between TYG-related indicators and outcome risk were more pronounced in the elderly, drinkers, and women (**Table 4**).

**Table 4:** Subgroup analyses for the association between TyG-related index and MCR in depressive population.

Subgroup	TyG-BMI	TyG-WC	TyG-WHtR	TyG
Gender				
woman	1.004 (1.000, 1.008) 0.0819	1.002 (1.001, 1.004) 0.0042	1.523 (1.192, 1.946) 0.0008	1.379 (0.999, 1.903) 0.0508
man	1.007 (1.001, 1.012) 0.0122	1.002 (1.0004, 1.0046) 0.0215	1.564 (1.103, 2.218) 0.0121	1.220 (0.799, 1.864) 0.3568
Age				
<60	0.990 (0.960, 1.020) 0.5038	0.998 (0.989, 1.008) 0.7489	0.710 (0.141, 3.582) 0.6779	0.056 (0.003, 1.245) 0.0685
>=60	1.003 (1.000, 1.007) 0.0595	1.002 (1.001, 1.003) 0.0016	1.546 (1.262, 1.895) <0.0001	1.285 (0.998, 1.654) 0.0521
Live status				
Urban Community	1.004 (0.999, 1.010) 0.0906	1.002 (1.0001, 1.0043) 0.0360	1.527 (1.098, 2.124) 0.0120	1.352 (0.909, 2.012) 0.1366
Rural Village	1.005 (1.001, 1.009) 0.0214	1.003 (1.001, 1.004) 0.0027	1.529 (1.188, 1.967) 0.0010	1.281 (0.915, 1.793) 0.1496
Smoke				
No	1.004 (1.0001, 1.0084) 0.0467	1.003 (1.001, 1.004) 0.0042	1.553 (1.195, 2.017) 0.0010	1.386 (0.992, 1.937) 0.0561
Yes	1.006 (1.0005, 1.0107) 0.0328	1.002 (1.0004, 1.0042) 0.0202	1.518 (1.109, 2.077) 0.0091	1.224 (0.822, 1.821) 0.3192
Drink				
No	1.003 (0.999, 1.007) 0.2018	1.002 (1.001, 1.004) 0.0108	1.438 (1.120, 1.847) 0.0044	1.380 (0.994, 1.915) 0.0541
Yes	1.008 (1.003, 1.013) 0.0020	1.003 (1.001, 1.005) 0.0090	1.692 (1.211, 2.364) 0.0021	1.197 (0.792, 1.811) 0.3934

## 4 Discussion

MCR is a prevalent and multifactorial clinical construct. According to a multinational study by Verghese et al., the global prevalence of MCR is approximately 9.7% (45). Its occurrence has been linked to diverse risk factors, including personality trait (46), obesity (47), smoking, low educational attainment, sedentary lifestyle, and depressive symptoms (48). Yuan et al. found that in rural Chinese older adults, low BMI was associated with MCI in women, whereas high BMI was more predictive in men (49). Obesity and its complications have been implicated in cognitive dysfunction, accelerated decline, and neurodegenerative disorders such as dementia (50), potentially through dysregulation of the hypothalamic-pituitary-adrenal (HPA) axis, altered cortisol rhythms, and structural changes in brain regions such as the hippocampus (51). Moreover, Li et al. reported that MCR was closely related to disturbances in triglyceride metabolism (52).

A prospective study by Beauchet et al. using the Canadian NuAge cohort demonstrated that individuals with both MCR and late-life depressive symptoms had a significantly increased risk of developing dementia (OR = 2.31; 95% CI: 1.51–3.52; P < 0.001), while either condition alone did not confer a significant risk. This suggests that depression and MCR may interact to accelerate cognitive deterioration (3). Similarly, Wang et al. found a stronger association between MCR and depression in females, aligning with our findings (53). Zhou et al. confirmed through meta-analyses that late-life depression significantly increases MCR risk (4).

In line with these findings, Bai et al. identified a significant association between elevated TyG index and cognitive impairment in older Chinese adults (54). Our study extends these results by demonstrating a robust nonlinear association between TyG-BMI and MCR. Supporting our findings, Zhang et al. showed that TyG-BMI is associated with cerebrospinal fluid levels of Aβ42 and Tau, hippocampal atrophy, and impaired cognition (55). Mediation analysis suggested that TyG-BMI may indirectly influence cognitive function through tau pathology and neurodegenerative mechanisms.

Notably, our study is the first to identify a nonlinear relationship between TyG-BMI and MCR in the CHARLS cohort. We observed a significant increase in MCR risk below the TyG-BMI threshold of 276.21, with risk plateauing beyond this value. This may reflect a saturation of the detrimental metabolic effects of IR and obesity on neurocognitive pathways, consistent with the metabolic threshold accumulation theory. which posits that cognitive risk accelerates until a saturation point is reached. This suggests that individuals with moderately elevated TyG-BMI— below extreme levels—may warrant particular clinical attention.

Subgroup analysis revealed that the association between TyG-related indices and MCR was most pronounced in older adults aged ≥60 years. Specifically, the TyG-WHtR index (OR = 1.546; 95% CI: 1.103–2.218; P = 0.012) exhibited statistically significant associations with MCR risk in this subgroup. These findings are consistent with previous evidence. Fanelli et al. emphasized that insulin resistance (IR)-related somatic conditions—such as obesity, type 2 diabetes, and hypertension—are significantly linked to impairments in cognitive domains, particularly fluid intelligence and processing speed (7). As composite indicators reflecting both glucose and lipid metabolism, TyG-related indices may capture key metabolic disturbances underlying IR-associated neurodegenerative processes. Older adults may be especially vulnerable to such mechanisms, including neuroinflammation, white matter degradation, and global metabolic dysregulation, which may account for the stronger associations observed in this group (**Table 4**).

The attenuated associations observed in individuals with higher educational attainment may reflect the protective role of cognitive reserve. According to this hypothesis, lifelong intellectual engagement—such as formal education, cognitively demanding occupations, and enriched environments—enhances neural efficiency, compensatory brain activation, and synaptic plasticity, thereby delaying the clinical manifestation of cognitive impairment even in the presence of metabolic disturbance (56–58). This neuroprotective effect may help explain why TyG-related indices were significantly associated with MCR only among less-educated individuals in our study.

Although all TyG-related indices demonstrated positive associations with MCR, only TyG-BMI exhibited a significant nonlinear threshold effect, as well as consistent and robust associations across multiple subgroups, including older adults, females, smokers, and individuals with lower educational attainment. These findings suggest that TyG-BMI may serve as a more sensitive and stable indicator of metabolic-cognitive risk, particularly in vulnerable populations. Given the global rise in late-life metabolic disorders and depression, our findings may inform international strategies for early cognitive risk screening using accessible metabolic markers like TyG-BMI.

The CES-D-10 scale used in this study has demonstrated good psychometric properties in numerous studies (59, 60), and is particularly suitable for depression screening in resource-limited settings. Some studies have shown that social isolation and loneliness significantly increase the incidence of depression (61–63). Future research could incorporate more comprehensive psychological measurement tools, such as assessments including social isolation (Lubben Social Network Scale) and loneliness (UCLA Loneliness Scale).

This study focuses on people with depressive symptoms, as depressive symptoms share biological pathways such as chronic low-grade inflammation and HPA axis dysregulation with cognitive decline and insulin resistance. This makes this population suitable for exploring the relationship between TyG index and MCR (54, 64, 65). The metabolic characteristics of the depressed population are special, and indicators such as TyG-BMI may have stronger predictive value in this group. The results of this study also verified the influence of depressive state on the relationship between TyG-BMI and MCR. In the future, it is recommended to expand to those without depressive symptoms and multi-country cohorts to test the universality of the results.

## 5 Conclusion

This study found a significant nonlinear association between TYG-BMI and the risk of MCR in the depressed population. As a comprehensive indicator of insulin resistance and obesity, TyG-BMI is of great significance to cognitive health. Its threshold effect indicates that early metabolic disorders are related to cognitive decline. Subgroup analysis emphasized the moderating roles of age and education. TyG-BMI is simple and easily accessible, and can serve as a practical tool for early identification of individuals at risk of cognitive impairment. Moreover, it can be adjusted through lifestyle and pharmacological interventions, and is clinically feasible. Future longitudinal and interventional studies can be conducted to determine whether improving TyG-BMI can reduce or delay the onset of MCR, which is crucial for advancing precision prevention methods for the aging population.

## Data Availability

The original contributions presented in the study are included in the article/**Supplementary Material**, further inquiries can be directed to the corresponding author/s.
