# Supplementary material for: Nonlinear association between TyG-related Indices and motoric cognitive risk syndrome in depressive individuals: evidence from the CHARLS study
Source: Front Psychiatry. 2025 Aug 28;16:1622973. doi: 10.3389/fpsyt.2025.1622973 (PMC12423043; doi:10.3389/fpsyt.2025.1622973)
Supplement: Supplementary file 1 [file Table1.docx]

Supplementary Material

# Supplementary Tables

**Supplementary table 1** Logistic regression analysis of the relationship between TyG-related index and MCR in depression population (CHARLS cohort study).

| **Exposure** | **Non-adjusted** | **Adjust I** | **Adjust II** |
| --- | --- | --- | --- |
| **TYGBMI** | 1.013 (1.0002, 1.0261) 0.04620 | 1.016 (1.002, 1.031) 0.02594 | 1.019 (1.004, 1.034) 0.01305 |
| **TYGWC** | 1.003 (0.999, 1.007) 0.16876 | 1.004 (0.999, 1.008) 0.11414 | 1.004 (0.999, 1.009) 0.09754 |
| **TYGWHTR** | 1.723 (0.919, 3.230) 0.08958 | 2.175 (1.041, 4.544) 0.03871 | 2.443 (1.124, 5.313) 0.02420 |
| **TYG** | 1.281 (0.633, 2.590) 0.49106 | 1.357 (0.644, 2.857) 0.42207 | 1.418 (0.660, 3.050) 0.37095 |

**Supplementary table 2** Logistic regression analysis of the relationship between TyG-related index and MCR in non-depression population (CHARLS cohort study)

| **Exposure** | **Non-adjusted** | **Adjust I** | **Adjust II** |
| --- | --- | --- | --- |
| **TYGBMI** | 1.001 (0.989, 1.014) 0.83966 | 1.003 (0.990, 1.016) 0.67771 | 1.004 (0.991, 1.017) 0.57041 |
| **TYGWC** | 0.999 (0.994, 1.003) 0.54472 | 0.999 (0.995, 1.004) 0.82582 | 1.000 (0.995, 1.004) 0.88420 |
| **TYGWHTR** | 0.862 (0.430, 1.726) 0.67505 | 0.825 (0.401, 1.698) 0.60074 | 0.848 (0.412, 1.745) 0.65420 |
| **TYG** | 0.861 (0.373, 1.989) 0.72690 | 0.845 (0.354, 2.015) 0.70351 | 0.863 (0.366, 2.034) 0.73575 |

**Supplementary table 3** Threshold Effect Analysis of TyG-BMI index on MCR Risk in depressed population (CHARLS cohort study).

| **Outcome** | **TYGBMI** |
| --- | --- |
| **Model I** | 1.019 (1.004, 1.034) 0.0131 |
| **Model II** |  |
| **Inflection point** | 190.155 |
| **< Inflection point** | 0.977 (0.940, 1.016) 0.2413 |
| **> Inflection point** | 1.033 (1.013, 1.054) 0.0011 |
| **Log-likelihood ratio test** | 0.035 |
